# The case for standardizing gene nomenclature in vertebrates

**DOI:** 10.1038/s41586-022-05633-w

**Published:** 2023-02-15

**Authors:** Fiona M. McCarthy, Tamsin E. M. Jones, Anne E. Kwitek, Cynthia L. Smith, Peter D. Vize, Monte Westerfield, Elspeth A. Bruford

**Affiliations:** 1 The Chicken Gene Nomenclature Committee (CGNC), School of Animal and Comparative Biomedical Sciences, University of Arizona, Tucson, AZ USA; 2 HUGO Gene Nomenclature Committee (HGNC), European Molecular Biology Laboratory, European Bioinformatics Institute, Wellcome Genome Campus, Hinxton, UK; 3 Rat Genome Database, Medical College of Wisconsin, Milwaukee, WI USA; 4 Mouse Genome Database, The Jackson Laboratory, Bar Harbor, ME USA; 5 Xenbase, Departments of Biological Sciences and Computer Science, University of Calgary, Calgary, Alberta Canada; 6 ZFIN, Institute of Neuroscience, University of Oregon, Eugene, OR USA; 7 Department of Haematology, University of Cambridge School of Clinical Medicine, Cambridge, UK

**Keywords:** Comparative genomics, Genome evolution, Phylogenomics

arising from C. Theofanopoulou et al. *Nature* 10.1038/s41586-020-03040-7 (2021)

Standardized gene nomenclature supports unambiguous communication and identification of the scientific literature associated with genes; to support the increasing number of annotated genomes that are now available for comparative studies, gene nomenclature authorities coordinate the assignment of approved gene names that can be readily applied across species. Theofanopoulou et al.^1^ propose a new nomenclature for the genes that encode oxytocin and arginine vasopressin and their receptors. Rather than changing to a different nomenclature, we suggest minor updates to the current approved nomenclature of these vertebrate genes to better reflect their evolutionary history. We call on authors, journal editors and reviewers to help support communication and indexing of gene-related publications by working with existing gene nomenclature committees and ensuring that standardized gene nomenclature is routinely used.

Standardized gene nomenclature provides a common language for the biomedical community and beyond. Gene nomenclature refers to both the full gene name and the unique gene symbol; aliases (or synonyms) used in published literature are also often recorded to facilitate disambiguation, indexing and text mining. In vertebrates, gene nomenclature committees focus on species that represent key groups, including mammals^2–4^, birds^5^, fish^6^ and amphibians^7^, and coordinate their efforts to ensure that approved gene names are assigned consistently across species. Historically, mammalian and avian gene symbols are in upper case, with the exception of rodents, which have gene symbols in title case; by contrast, *Xenopus* and zebrafish gene symbols are in lower case. These case conventions were originally established to help researchers distinguish references to genes in different model organisms^8^, and the standardized nomenclature is widely disseminated through all of the major genomic resources and model organism databases. Notably, this approach takes into account genetic and evolutionary similarities in addition to function, exactly as proposed by Theofanopoulou et al.^1^, and many genes are named on the basis of their homologues in yeast, flies and other non-vertebrates. Gene nomenclature groups work closely with community experts^9^, researchers, clinicians, bioinformaticians and biocurators to ensure that the approved gene names and symbols are informative, non-redundant and broadly applicable across diverse biological fields of study. One rationale cited for the newly proposed nomenclature of Theofanopoulou et al. is to create a universal nomenclature system that can be consistently used across vertebrates. However, such a system is already established by the existing vertebrate nomenclature authorities.

Theofanopoulou et al. propose a new nomenclature for the genes that encode oxytocin, arginine vasopressin and their receptors on the basis of their evolutionary analysis of these genes in the context of newly sequenced, high-quality genomes generated by the Vertebrate Genomes Project (VGP)^10^. Although we share their desire to ensure that gene nomenclature reflects evolutionary relationships, we believe that the existing approved nomenclature, first established in vertebrates 30 years ago, already largely represents these relationships (Table 1). Instead, only minor updates are needed in some species to better reflect the orthology and paralogy between these genes (Supplementary Information). We consider many factors when making nomenclature decisions: structure and function of genes and gene products; evolutionary history (including consideration of gene synteny); current and historical nomenclature usage; utility of nomenclature as search terms (including avoiding symbol clashes with other genes across the tree of life); levels of support for nomenclature updates in the research community; and concordance with nomenclature guidelines in several model systems (see Supplementary Information). In addition, the current remit of the HUGO Gene Nomenclature Committee (HGNC) includes a commitment to move towards gene-symbol stability in humans^2^—especially for genes that are clinically relevant, which include the genes encoding oxytocin and arginine vasopressin and their receptors. Confusion about gene nomenclature in the medical literature could have serious negative consequences for patient safety^11^.

**Table 1:** Comparison of approved and proposed symbols for the oxytocin and arginine vasopressin ligand and receptor genes

Approved symbol from joint nomenclature committees	Theofanopoulou et al. proposed symbol
*OXT*	*OT*
*AVP*	*VT*
*OXTR*	*OTR*
*AVPR1A*	*VTR1A*
*AVPR1B*	*VTR1B*
*AVPR2* (aliased as AVPR2A*)	*VTR2C*
AVPR2B*	*VTR2B*
AVPR2C* or *AVPR2L*	*VTR2A*

Newly approved symbols are indicated with *.

Major revisions to approved nomenclature are considered when the benefits clearly outweigh the downsides. Benefits can include the correction of incorrect or misleading gene nomenclature, better representation of evolutionary relationships, standardizing nomenclature throughout a gene family and providing nomenclature that can be used across all vertebrate species. Theofanopoulou et al. argue that the nomenclature of the oxytocin and arginine vasopressin genes and their receptors merits an update for all of these benefits. We believe that the existing approved nomenclature does not merit major revision as it is widely used, is not incorrect or misleading in the vast majority of vertebrate species, largely represents evolutionary relationships (with only minor additions needed to represent subclades in the AVPR2 subfamily) and has long been standardized across species (see Supplementary Information). The drawback is the introduction of additional identifiers in databases and the literature, which increases the risk of confusion to researchers and readers. Unfortunately, the potential for confusion has already been exemplified in a recent paper by Ocampo Daza et al.^12^, who disagreed with Theofanopoulou et al.’s assignment of ABC suffixes in the AVPR2/VTR2 subfamily and therefore used the same symbols to refer to different genes.

Theofanopoulou et al. argue that their study acts as a model for the revision of gene nomenclature in the context of large-scale vertebrate-sequencing projects, including the VGP. Their stated intent is to completely revise vertebrate gene nomenclature, including that of human genes, to fully reflect evolutionary histories that are revealed by large-scale sequencing projects. We are concerned that the authors may not fully appreciate the level of disruption that would be caused by major revisions to gene nomenclature on this scale. It is worth noting that the gene family analysed in their study is relatively simple with regard to its evolutionary history, and to perform such an analysis for every vertebrate gene family is an inconceivably large task. Given that over 40 years and millions of dollars of public funding have been invested in the current standardized nomenclature projects, we propose that an overhaul of the entire system would not be a prudent use of the limited resources we have in genomics.

Requiring scientists to consistently use approved nomenclature avoids confusion and supports search indexing. Although an increasing number of scientific journals mandate the use of standardized gene nomenclature, this requirement is not always clearly stated or strictly enforced for authors, and citing the approved gene symbol and its associated gene ID should be compulsory in all journals. The instructions provided by *Nature* to authors state that authors can “use their preferred terminology” for genes and proteins, which enables authors to publish novel nomenclature without first checking with the relevant authority. If all journals—and especially influential ones such as *Nature*—were to insist that authors consult with nomenclature committees when suggesting updates, much confusion could potentially be avoided. Unequivocally communicating about genes will facilitate research and development in all biological and clinical fields.

We assert that the changes suggested by Theofanopoulou et al. to the official vertebrate gene nomenclature would cause considerable confusion with little perceivable benefit.

Our analysis of their study (Supplementary Information) demonstrates how the integration of genomic data from a broader range of species can help us to update and improve an already-established nomenclature with only minor modifications. Theofanopoulou et al. call for collaboration between the gene nomenclature committees and genomic initiatives, which we have always wholeheartedly supported. We continue to encourage researchers and communities to collaborate with the gene nomenclature committees when proposing nomenclature updates.

## Reporting summary

Further information on experimental design is available in the Nature Portfolio Reporting Summary linked to this article.

## Supplementary information

Supplementary Information This file contains additional discussion of the guidelines and procedures used by the gene nomenclature committees, followed by detailed discussion of the nomenclature of each gene in the oxytocin/arginine vasopressin ligand and receptor gene families. Supplementary Tables 1–5 outline the key factors considered by the gene nomenclature committees when deciding whether to update approved gene nomenclature. Supplementary Tables 6–13 specify the approved gene nomenclature and unique database IDs for all genes as well as indicating where the approved nomenclature was updated concurrently with this publication. Supplementary Fig. 1 shows a maximum likelihood phylogeny of the vertebrate AVPR2* amino acid sequences.

Reporting Summary
