# Supplementary material for: The case for standardizing gene nomenclature in vertebrates
Source: Nature. 2023 Feb 15;614(7948):E31–2. doi: 10.1038/s41586-022-05633-w (PMC9931569; doi:10.1038/s41586-022-05633-w)
Supplement: Supplementary file 1 — This file contains additional discussion of the guidelines and procedures used by the gene nomenclature committees, followed by detailed discussion of the nomenclature of each gene in the oxytocin/arginine vasopressin ligand and receptor gene families. Supplementary Tables 1–5 outline the key factors considered by the gene nomenclature committees when deciding whether to update approved gene nomenclature. Supplementary Tables 6–13 specify the approved gene nomenclature and unique database IDs for all genes as well as indicating where the approved nomenclature was updated concurrently with this publication. Supplementary Fig. 1 shows a maximum likelihood phylogeny of the vertebrate AVPR2* amino acid sequences. [file 41586_2022_5633_MOESM1_ESM.docx]

**Supplementary Information - The Case for Standardising Gene Nomenclature in Vertebrates**

Gene nomenclature committees and abbreviations:

CGNC: Chicken Gene Nomenclature Committee

HGNC: HUGO Gene Nomenclature Committee

MGNC: Mouse Genome Nomenclature Committee

RGD: Rat Genome Database

Xenbase: The Xenopus model organism database

VGNC: Vertebrate Gene Nomenclature Committee

ZNC: Zebrafish Nomenclature Committee

***Naming genes across vertebrates***

The gene nomenclature committees use multiple streams of evidence when approving gene nomenclature. We routinely consider gene synteny, phylogenetic inference and ortholog calls from multiple public resources to identify homologous relationships between genes and between genes from different species. We also read published literature about the gene, contact the authors proposing new gene nomenclature and consult experts who have published on specific gene families. However, we do not have blanket “rules” about which factors should be weighed more heavily than others, as each case will differ depending on the context: the levels of evolutionary complexity, availability of functional information, nomenclature history, etc., vary widely from gene to gene and family to family. Proposed updates to approved nomenclature are considered carefully, taking into account multiple factors to decide whether a nomenclature update would improve communication about the gene(s). A matrix exemplifying some key factors we consider is shown in Table S1.

**Table S1. Key factors considered by the gene nomenclature committees when deciding whether to update approved gene nomenclature.**

|  | Factors in favour of a nomenclature update | Factors in favour of retaining existing nomenclature system |
| --- | --- | --- |
| Is the current approved nomenclature incorrect or misleading? | YES | NO |
| Is the current approved nomenclature causing confusion in the literature? | YES | NO |
| Is the current approved nomenclature NOT being widely used by researchers? | YES | NO |
| Would a nomenclature update significantly improve communication about the gene? | YES | NO |
| Is the current nomenclature NOT a good, unique search term? | YES | NO |
| Does the current nomenclature NOT reflect evolutionary relationships? | YES | NO |

Gene nomenclature committees devote particular attention and manual curation efforts to the nomenclature of gene families (and other gene groups such as genes with shared function), often enlisting the invaluable help of community experts (“specialist advisors” e.g. https://www.genenames.org/about/specialist-advisors/), where possible. In many gene families there are substantial expansions and contractions between species which require careful disambiguation so that homologs and species-specific genes are correctly identified and named. The gene nomenclature committees work together to coordinate these efforts and ensure that newly proposed nomenclature will be suitable for use across all vertebrates. These efforts can be very time intensive, sometimes requiring dedicated funding to facilitate novel analyses such as phylogenetic and synteny analyses to establish homology relationships, as exemplified by Theofanopoulou et al.’s study [(Theofanopoulou et al. 2021)](https://paperpile.com/c/qR92DB/30ir) on the OXT and AVP receptor genes. We are aware that there remain many gene families that could benefit from dedicated efforts to improve gene nomenclature and always welcome collaboration with specialist researchers. A major difference between our approach and that of Theofanopoulou et al. is that we consult with the research community who publish on the gene family of interest, not only to solicit their feedback but also to raise awareness of potential nomenclature updates in the hope that new nomenclature will be implemented in future publications.

The most commonly used identifiers for genes in the literature are gene symbols, making the stability of gene symbols a high priority to facilitate the identification of literature about specific genes. A proposed gene symbol should be long enough that it can be distinct and readily used to search literature - two letter symbols (e.g., VT and OT) are discouraged as they are likely to be used in the literature for a range of confounding abbreviations. Researchers working in text mining and indexing routinely request that gene symbols are longer than three characters to facilitate disambiguation. Conversely, increasingly longer and more complicated symbols are not always easily memorable. With the growth of scientific literature and the increasing importance of indexing and machine learning, the need for unique identifiers has increased importance and weighting in gene symbol assignment. Nomenclature committees assign unique identifiers (eg. HGNC:#####, ZFIN:ZDB-GENE-######) for cross-referencing but people prefer gene symbols. Every gene symbol should be unique, and as we assign new gene symbols we strive to avoid symbols that are used for other genes (at least across vertebrates, but invertebrate and even bacterial genes are also considered).

Synonyms or aliases are used to capture additional names that have been used in the literature for a particular species to help with literature indexing and searching. To clarify, genes are assigned a standardized gene name and symbol which should be the primary method for referring to genes, and the synonyms (for example, those displayed in NCBI Gene as “Also known as”) are meant simply to assist with mining literature and databases. Gene synonyms (or aliases) are not recommended for routine use but are captured to support data mining and indexing.

We note that many journals have guidelines that specifically state for authors to use gene nomenclature in accordance with established conventions, where a gene nomenclature authority exists. As Theofanopoulou et al. point out, not all vertebrate species have such a committee. Developing a gene nomenclature authority requires sustained funding and resources that are only available for a limited number of species. For example, a nomenclature committee for the Anole lizard was formed [(Kusumi et al. 2011)](https://paperpile.com/c/qR92DB/g9VG), but has not been active since the initial publication and thus this species still lacks standardized nomenclature. Following efforts by the HGNC to extend standardized nomenclature to more vertebrate species, the Vertebrate Gene Nomenclature Committee was established [(Yates et al. 2017)](https://paperpile.com/c/qR92DB/iyVj) and now approves nomenclature in 7 key mammalian species [(Tweedie et al. 2021)](https://paperpile.com/c/qR92DB/jo19), with plans to expand to further vertebrate species where funding allows. Nonetheless, existing nomenclature committees routinely include other species in their analyses to ensure that approved nomenclature is suitable to be automatically propagated across species (as provided in the NCBI and Ensembl databases, for example). All vertebrate gene nomenclature committees actively collaborate with NCBI curators to extend gene symbols and names to vertebrate species not specifically represented by a nomenclature group.

There are many instances where an overhaul to an entire nomenclature system would be impractical and disruptive but minor changes to gene names can be implemented where necessary. For example, a 2021 publication [(Malatesta et al. 2020)](https://paperpile.com/c/qR92DB/BJvw) provided evidence in chickens that the *CSAD* “cysteine sulfinic acid decarboxylase” gene (NCBI Gene ID: 426184) protein product had cysteine acid decarboxylase activity but not cysteine sulfinic acid decarboxylase activity, due to two amino acid substitutions. After consultation with the authors, the chicken *CSAD* symbol was retained to indicate this gene’s evolutionary relationship but the gene name was changed to “cysteine acid decarboxylase”. Researchers and clinicians overwhelmingly choose to use gene symbols rather than full gene names in their publications. By keeping the gene symbol the same across species, it is clear that these genes in different species are orthologs. Making changes to a gene name to indicate changes of structure or function that have arisen over its evolutionary history allows biologists to note these differences without compromising the link to the large number of studies and resources that rely on stable gene symbols.

***Standardized nomenclature for oxytocin and arginine vasopressin***

Oxytocin is a well-studied peptide hormone and neuropeptide with a large body of published scientific literature. The human gene name ‘oxytocin/neurophysin I prepropeptide’ with the symbol *OXT* (HGNC:8528) represents the full length protein which is post-translationally cleaved to produce oxytocin and neurophysin I, the oxytocin carrier protein [(Brownstein, Russell, and Gainer 1980)](https://paperpile.com/c/qR92DB/Jxn1). We agree with Theofanopoulou et al. that the terms “mesotocin” and “isotocin”, used for avian and fish orthologs respectively, should be retired as their use obscures the high level of conservation of oxytocin genes across the vertebrates. An important feature of gene symbols is that they should be specific search terms. The “OT” symbol proposed by Theofanopoulou et al. returns over 12,000 PubMed results, many which are not related to oxytocin (or genes), making it a poor search term.

The arginine vasopressin gene (with the symbol *AVP,* HGNC:894) encodes a preprotein that is cleaved to form arginine vasopressin, neurophysin II and copeptin [(Brownstein, Russell, and Gainer 1980; Land et al. 1982)](https://paperpile.com/c/qR92DB/Jxn1+7jb96). Because of the action of these peptide hormones as antidiuretics and vasoconstrictors, this gene is well studied with a body of literature that has now settled on a common gene name for vertebrate orthologs, including Xenopus and zebrafish. Theofanopoulou et al*.* suggest that vasotocin is commonly used, however a PubMed search returns only 2,581 results for ‘vasotocin’, compared to 48,281 results for ‘vasopressin’. Furthermore, the approved name ‘arginine vasopressin’ refers to a highly conserved arginine in the AVP peptide product, which is present in the vast majority of sequenced vertebrates.

The existing approved *OXT* and *AVP* root symbols have been approved in human since the early 1990s, and propagated to other vertebrates subsequently. Changing these to the proposed two letter symbols would only result in confusion and hinder literature searches. Gene symbol stability is especially important for genes that are linked to human health, and the oxytocin and vasopressin ligands and receptors all fall into this category, with thousands of papers using the current approved nomenclature.

***Standardized nomenclature for oxytocin and arginine vasopressin receptors***

Theofanopoulou et al*.* have confirmed the existence of six distinct clades of the oxytocin/vasopressin receptor family in vertebrates and proposed a novel nomenclature system for these clades. While we share their desire to ensure gene nomenclature reflects evolutionary relationships, we disagree that there is a need to revise all of the currently approved gene symbols to achieve this, as the existing approved nomenclature system is already largely representing these relationships (Tables S6-S13), and the AVPR root symbol has been approved across vertebrates for many years. Instead, only minor updates are needed in some species to better reflect the orthology and paralogy between these genes.

We also disagree with the stated order of gene divergence presented by Theofanopoulou et al. for the AVPR2 clade. The authors state that their phylogenetic findings are consistent with their synteny analysis and conclude that *AVPR2C* (which they refer to as *VTR2A*) first diverged from the common ancestor of *AVPR2* and *AVPR2B* (*VTR2C* and *VTR2B*, respectively). However, the phylogenies presented in their publication show that the *AVPR2* gene first diverged from the common ancestor of the *AVPR2C* and *AVPR2B* genes prior to the duplication that gave rise to the *AVPR2C* and *AVPR2B* clades. This suggests that, despite its absence in sharks, AVPR2 may have been present in the common ancestor of vertebrates and was subsequently lost in some lineages, including sharks, conflicting with the synteny analysis and ultimate conclusions reached by Theofanopoulou et al. We do not attempt to resolve this evolutionary history, as we assign each of the three major clades unique nomenclature regardless of their order of evolutionary divergence. We posit that the least disruptive change to make to the approved nomenclature is to retain the current mammalian symbol for *AVPR2* (with an alias of *AVPR2A*) and transfer this symbol to its orthologs. We therefore propose to use the same root symbol (AVPR) for its paralogs, appending the letters B and C, as shown in Tables S12-S13. Theofanopoulou et al. propose to rename *AVPR2* as *VTR2C* (and use *VTR2A* for the paralog that we would approve as *AVPR2C*), which we believe would be highly confusing to researchers. Additionally, a recent study by Ocampo Daza et al. [(Ocampo Daza, Bergqvist, and Larhammar 2021)](https://paperpile.com/c/qR92DB/KjhK) made the decision to swap the “A” and “C” suffixes with respect to Theofanopoulou et al.’s assignment (that is, they use *VTR2A* for the genes currently approved as *AVPR2(A),* and *VTR2C* for the genes now approved as *AVPR2C*), which, while remaining more in line with the existing usage of these suffixes in the literature, unfortunately contributes to yet another potential source of confusion about which suffix is used for which paralog.

There is some uncertainty about whether teleost *avpr2l* genes are orthologous to *AVPR2C* genes in other taxa, despite their partial shared synteny. Reciprocal BLAST searches and phylogenetic analysis (Figure S1) do not group *avpr2l* with *AVPR2C* genes. Although both Theofanopoulou et al*.* and the more recent study by Ocampo Daza et al*.* [(Ocampo Daza, Bergqvist, and Larhammar 2021)](https://paperpile.com/c/qR92DB/KjhK) conclude that shared synteny is sufficient to determine that teleost *avpr2l* and *AVPR2C* in other taxa are identical by descent, these studies do not agree on *avpr2l*’s placement in phylogenetic analyses. When there is disagreement among methods about the evolutionary history of a gene, we prefer to be conservative when assigning nomenclature as the potential for confusion is higher when genes that have the same symbol are later found to not be 1:1 orthologs. Due to the uncertainty about the lineage of *avpr2l*, we have not updated this nomenclature in zebrafish.

**1. Oxytocin gene nomenclature**

**Table S2. Key factors considered by the gene nomenclature committees when deciding whether to update approved gene nomenclature for *OXT*.**

|  | Factors in favour of a nomenclature update | Factors in favour of retaining existing nomenclature system | Notes |
| --- | --- | --- | --- |
| Is the current approved nomenclature incorrect or misleading? | YES | NO  ✔ |  |
| Is the current approved nomenclature causing confusion in the literature? | YES | NO  ✔ |  |
| Is the current approved nomenclature NOT being widely used by researchers? | YES | NO  ✔ |  |
| Would a nomenclature update significantly improve communication about the gene? | YES | NO  ✔ |  |
| Is the current nomenclature NOT a good, unique search term? | YES | NO  ✔ |  |
| Does the current nomenclature NOT reflect evolutionary relationships? | YES  ✔ | NO | Nomenclature for *OXT* and *AVP* does not reflect their paralogous relationship, however their names are so well-established that a change would likely cause considerable confusion. |

Table S2 shows the key factors considered by the gene nomenclature committees when deciding whether to update the approved gene nomenclature for *OXT*. The existing approved nomenclature (Table S6) accurately represents the orthologous relationships between the genes across species. The current *OXT* symbol is widely used in the literature (1,194 results in PubMed) and highly specific – that is, its use as a search term accurately identifies publications mentioning the oxytocin gene.

The proposed symbol by Theofanopoulou et al. [( 2021)](https://paperpile.com/c/qR92DB/30ir), OT, in contrast, is not a specific search term. While it is in use by many papers in the literature to refer to the oxytocin gene, a PubMed search for “OT” returns over 25,000 hits, less than 15% of which refer to oxytocin. Clashes include acronyms for terms such as “operative time”, “occupational therapy”, and the much studied OT-I/II transgenic mice.

We have retained the existing approved nomenclature for oxytocin as shown in Table S6. OT should be included as an “alias” symbol in databases since it is used in the literature, but we encourage researchers to use the approved gene nomenclature to ensure easy identification of their papers and minimise confusion.

**2.** **Arginine vasopressin gene nomenclature**

**Table S3. Key factors considered by the gene nomenclature committees when deciding whether to update approved gene nomenclature for *AVP*.**

|  | Factors in favour of a nomenclature update | Factors in favour of retaining existing nomenclature system | Notes |
| --- | --- | --- | --- |
| Is the current approved nomenclature incorrect or misleading? | YES | NO  ✔ | Gene names have been updated to reflect the absence of arginine in marsupials and suids. |
| Is the current approved nomenclature causing confusion in the literature? | YES | NO  ✔ |  |
| Is the current approved nomenclature NOT being widely used by researchers? | YES | NO  ✔ |  |
| Would a nomenclature update significantly improve communication about the gene? | YES | NO  ✔ |  |
| Is the current nomenclature NOT a good, unique search term? | YES | NO  ✔ |  |
| Does the current nomenclature NOT reflect evolutionary relationships? | YES  ✔ | NO | Nomenclature for *OXT* and *AVP* does not reflect their paralogous relationship, however their names are so well-established that a change would likely cause confusion. |

Table S3 shows the key factors considered by the gene nomenclature committees when deciding whether to update the approved gene nomenclature for AVP. The existing approved nomenclature (Table S7) accurately represents the orthologous relationships between the genes across species. The current symbol *AVP* is widely used in the literature (10,658 hits in PubMed) and is highly specific, with around 88% of PubMed hits specifically referring to the gene/gene product. The proposed symbol by Theofanopoulou et al*.* (2021), VT, in contrast, is not a specific search term and has not been widely used in the literature to refer to this gene. A PubMed search for “VT” returns over 37,000 results, only 227 of which mention “vasotocin” or “vasopressin”.

One drawback to the current approved nomenclature that was raised by Theofanopoulou et al. is the inclusion of “arginine” in the gene name, which refers to a highly conserved amino acid in the AVP peptide product that is not present in all vertebrates. We have identified only two lineages in which this arginine residue is absent: suidae and marsupials. To avoid confusion in the literature, and in biological databases where the existing approved gene nomenclature has already propagated, we will retain the existing symbols but modify the gene names in the species where the arginine residue is not present to “vasopressin” to avoid any confusion. Of the species with approved nomenclature, this currently only affects the pig *Sus scrofa*. We have retained all other aspects of the existing approved nomenclature for arginine vasopressin as shown in Table S7. VT should be included as an “alias” symbol in databases since it is used in the literature, but we encourage researchers to use the approved gene nomenclature to ensure easy identification of their papers and minimise confusion.

**3.** **Oxytocin receptor gene nomenclature**

**Table S4. Key factors considered by the gene nomenclature committees when deciding whether to update approved gene nomenclature for *OXTR*.**

|  | Factors in favour of a nomenclature update | Factors in favour of retaining existing nomenclature system | Notes |
| --- | --- | --- | --- |
| Is the current approved nomenclature incorrect or misleading? | YES | NO  ✔ |  |
| Is the current approved nomenclature causing confusion in the literature? | YES | NO  ✔ |  |
| Is the current approved nomenclature NOT being widely used by researchers? | YES | NO  ✔ |  |
| Would a nomenclature update significantly improve communication about the gene? | YES | NO  ✔ |  |
| Is the current nomenclature NOT a good, unique search term? | YES | NO  ✔ |  |
| Does the current nomenclature NOT reflect evolutionary relationships? | YES | NO  ✔ | The two zebrafish genes have been named to reflect that they are co-orthologs (Table S8). |

Table S4 shows the key factors considered by the gene nomenclature committees when deciding whether to update the approved gene nomenclature for OXTR. The existing approved nomenclature (Table S8) accurately represents both the function of the gene(s), and the orthologous relationships between the genes across species. The current *OXTR* symbol is widely used in the literature (928 results in PubMed) and highly specific – that is, its use as a search term accurately identifies publications mentioning the oxytocin receptor gene.

The proposed symbol by Theofanopoulou et al*.* (OTR), in contrast, is not a specific search term. While it is in use by many papers in the literature to refer to the oxytocin receptor gene, it returns over 3,000 hits in PubMed, most of which refer to the abbreviation of a journal name (*Ortop*. *Traumatol*. *Rehabil*.). Other results returned for the OTR acronym include “organ transplant recipients”, “oxygen transfer rate”, “ocular tilt reaction”, and “OXPHOS transcriptional response”.

We have retained the existing nomenclature system for *OXTR* genes but have made minor updates to the zebrafish genes (Table S8). In line with the findings of Theofanopoulou et al*.* we have changed the zebrafish gene nomenclature to *oxtra* and *oxtrb* (with correspondingly updated gene names) and retained the existing approved nomenclature for other species. OTR should be included as an “alias” symbol in databases since it is used in the literature, but we encourage researchers to use the approved gene nomenclature to ensure easy identification of their papers and minimise confusion.

**4. Arginine vasopressin receptors**

**Table S5. Key factors considered by the gene nomenclature committees when deciding whether to update approved gene nomenclature for the *AVPR* genes.**

|  | Factors in favour of a nomenclature update | Factors in favour of retaining existing nomenclature system | Notes |
| --- | --- | --- | --- |
| Is the current approved nomenclature incorrect or misleading? | YES | NO  ✔ | Gene names have been updated to reflect the absence of arginine in marsupials and suids. |
| Is the current approved nomenclature causing confusion in the literature? | YES | NO  ✔ |  |
| Is the current approved nomenclature NOT being widely used by researchers? | YES | NO  ✔ |  |
| Would a nomenclature update significantly improve communication about the gene? | YES | NO  ✔ |  |
| Is the current nomenclature NOT a good, unique search term? | YES | NO  ✔ |  |
| Does the current nomenclature NOT reflect evolutionary relationships? | YES | NO  ✔ | Additions have been made to the approved nomenclature to represent additional clades of AVPR2* genes, outlined below, and shown in Tables S12 and S13. |

Table S5 shows the key factors considered by the gene nomenclature committees when deciding whether to update the approved gene nomenclature for the AVPR genes.

***a. AVPR1A* (referred to as VTR1A in Theofanopoulou et al*.*)**

The existing approved nomenclature (Table S9) accurately reflects the orthologous relationships between the genes across species. It is used in the literature and is a specific search term for this gene.

As Theofanopoulou et al*.* state, the drawback of the current approved nomenclature is that in some species it has become clear that the “arginine” residue referenced in the gene name is not present, and thus it may be misleading in those species. To avoid confusion in the literature, and in biological databases where the existing approved gene nomenclature has already propagated, we have retained the existing symbols but modify the gene names in the species where the arginine residue is not present to “vasopressin receptor” to avoid any confusion. Of the species with approved nomenclature, this currently only affects pig. It also affects some marsupials, and the corresponding gene names can be modified when these species receive official nomenclature.

**b. *AVPR1B* (referred to as VTR1B in Theofanopoulou et al*.*)**

The existing approved nomenclature system (Table S10) accurately reflects the orthologous relationships between the genes across species. The symbol is used in the literature and is a specific search term for this gene.

As for other AVP* gene names, we have removed the word “arginine” from the gene name in pig, while retaining the symbol. We have also updated the gene name in rat to bring it in line with the gene name used by the other gene nomenclature committees. These updates are shown in Table S10. We will retain the existing nomenclature for all other species.

**c*. AVPR2* (referred to as VTR2C in Theofanopoulou et al*.*)**

The existing approved nomenclature (Table S11) accurately reflects the orthologous relationships between the genes across species. It is used in the literature and is a specific search term for this gene.

We recommend the retention of this symbol as it is currently approved, while aliasing the mammalian and Xenopus symbols as “AVPR2A”. This allows the current symbols to be retained, minimizing disruption, while also encoding the orthology relationships to the zebrafish genes, which already contain the “a” in their symbols (Table S11).

As in other AVP* gene names, we have removed the word “arginine” from the gene name in pig, while retaining the symbol. We have also updated the gene name in Xenopus to bring it in line with the gene name used by the other gene nomenclature committees. These updates are shown in Table S11. We will retain the existing nomenclature for all other genes.

**d. *AVPR2B* (referred to as VTR2B in Theofanopoulou et al*.*)**

This gene is absent from all but one of the species with approved nomenclature. Zebrafish has only one copy of this gene (ZFIN:ZDB-GENE-131127-163) but other teleost fish have two, so one of the copies was likely lost in the lineage giving rise to zebrafish. For this reason, Theofanopoulou et al*.* propose assigning the symbol “*vtr2ba*” to this gene, i.e., “vasotocin receptor 2b, duplicate a” (although they also refer to it as “VTR2Bb” in Figure 4a, the supplementary information indicates that *vtr2ba* is the proposed symbol). We have renamed it as *avpr2b.1* (arginine vasopressin receptor 2b, tandem duplicate, 1) to retain the root symbol that is already in use across vertebrates (Table S12) and to conform with zebrafish gene nomenclature guidelines [(Bradford et al. 2022)](https://paperpile.com/c/qR92DB/OtYA).

**e. *AVPR2C* / *avpr2l* (referred to as VTR2A in Theofanopoulou et al*.*)**

Although Theofanopoulou et al*.* propose the use of the “A” letter in their nomenclature system for this set of genes, we believe the least disruptive option is to reserve “A” for the orthologs of the human gene *AVPR2* (HGNC:897). This remains consistent with the way A/B/C suffixes have already been used for genes in the AVPR2* family, and ensures that the most well studied genes retain their approved nomenclature while necessary changes are applied to paralogs that have received less attention in the literature thus far.

Since CGNC:7225 is the only paralog of *AVPR2* that chicken has retained, it has been historically named in line with the human *AVPR2*, though it is not a direct ortholog [(Ocampo Daza, Lewicka, and Larhammar 2012; Yamaguchi et al. 2012)](https://paperpile.com/c/qR92DB/5caD+6ER3). We have updated the approved nomenclature of this gene to *AVPR2C*, and also updated the symbol of the Xenopus ortholog to *avpr2c* (Table S13).

Theofanopoulou et al. propose that the teleost fish genes currently referred to as *avpr2l* are orthologs of the *AVPR2C* genes, due to partial shared synteny with the *AVPR2C* genes in birds, reptiles, amphibians and non-teleost fish. We are unable to find phylogenetic support for this hypothesis (Fig. S1). The Avpr2l amino acid sequences do not cluster with any of the other three AVPR2* clades with strong support in our phylogenetic analysis. A similar study recently conducted by Ocampo Daza et al*.* [(Ocampo Daza, Bergqvist, and Larhammar 2021)](https://paperpile.com/c/qR92DB/KjhK) (in which these genes are labelled *VTR2C*) does not show these two clades as monophyletic. Despite their differing phylogenetic results, both Theofanopoulou et al*.* and Ocampo Daza et al*.* conclude that the synteny analysis supports these genes being orthologous. The position of the gene nomenclature committees is generally to be conservative when there are conflicting data about orthology, and rather than prioritizing one method over another, we prefer to retain the existing approved nomenclature while such conflicts exist. This reasoning is rooted in our experience of conflicting analyses leading to multiple changes in gene nomenclature over time, causing considerable confusion in the literature. Further, zebrafish has only one copy of this gene and it is not fully syntenic with its orthologs in the other teleost fish species examined, leading Theofanopoulou et al*.* to propose that it represents a duplication of the ancestral gene in teleosts (ie. that it is an out-paralog with respect to the *avpr2l* genes in other teleost species) and that it be named “VTR2Ab” with the other fish orthologs being “VTR2Aa”. We find no evidence that these genes are not in single copy in teleost fish. For these reasons we have left the nomenclature of zebrafish *avpr2l* unchanged.

The approved nomenclature of the *AVPR2C* and *avpr2l* genes is shown in Table S13.

**Table S6:** Approved nomenclature for the oxytocin genes in vertebrates.

| **Gene Nomenclature Committee** | **Species** | **Database unique ID** | **Current Approved Symbol** | **Current Approved Name** |
| --- | --- | --- | --- | --- |
| HGNC | human | HGNC:8528 | *OXT* | oxytocin/neurophysin I prepropeptide |
| MGNC | mouse | MGI: 97453 | *Oxt* | oxytocin |
| RGD | rat | RGD:3238 | *Oxt* | oxytocin/neurophysin I prepropeptide |
| VGNC | chimpanzee, macaque, cat, dog, horse, cow, pig | VGNC:6224, VGNC:75730, VGNC:108054, VGNC:54339, VGNC:108055, VGNC:32515, VGNC:96466 | *OXT* | oxytocin/neurophysin I prepropeptide |
| CGNC | chicken | CGNC:13728 | *OXT* | oxytocin/neurophysin I prepropeptide |
| Xenbase | Xenopus | Xenbase:XB-GENE-478274 | *oxt* | oxytocin/neurophysin I prepropeptide |
| ZNC | zebrafish | ZFIN:ZDB-GENE-030407-1 | *oxt* | oxytocin |

**Table S7:** Approved nomenclature for the arginine vasopressin genes in vertebrates. Newly approved nomenclature is highlighted in bold.

| **Gene Nomenclature Committee** | **Species** | **Database unique ID** | **Current Approved Symbol** | **Current Approved Name** |
| --- | --- | --- | --- | --- |
| HGNC | human | HGNC:894 | *AVP* | arginine vasopressin |
| MGNC | mouse | MGI:88121 | *Avp* | arginine vasopressin |
| RGD | rat | RGD:2184 | *Avp* | arginine vasopressin |
| VGNC | chimpanzee, macaque, cat, dog, horse, cow, pig | VGNC:6048, VGNC:107771, VGNC:98511, VGNC:38317, VGNC:58944, VGNC:26356, VGNC:96482 | *AVP* | arginine vasopressin  (**vasopressin** in pig only) |
| CGNC | chicken | CGNC:10532 | *AVP* | arginine vasopressin |
| Xenbase | Xenopus | Xenbase:XB-GENE-478869 | *avp* | arginine vasopressin |
| ZNC | zebrafish | ZFIN:ZDB-GENE-030407-2 | *avp* | arginine vasopressin |

**Table S8:** Approved nomenclature for the oxytocin receptor genes in vertebrates. Newly approved nomenclature is highlighted in bold.

| **Gene Nomenclature Committee** | **Species** | **Database unique ID** | **Current Approved Symbol** | **Previous Approved Symbol** | **Current Approved Name** | **Previous Approved Name** |
| --- | --- | --- | --- | --- | --- | --- |
| HGNC | human | HGNC:8529 | *OXTR* |  | oxytocin receptor |  |
| MGNC | mouse | MGI:109147 | *Oxtr* |  | oxytocin receptor |  |
| RGD | rat | RGD:3239 | *Oxtr* |  | oxytocin receptor |  |
| VGNC | chimpanzee, macaque, cat, dog, horse, cow, pig | VGNC:12118, VGNC:75731, VGNC:68667, VGNC:44206, VGNC:21105, VGNC:32516, VGNC:108052 | *OXTR* |  | oxytocin receptor |  |
| CGNC | chicken | CGNC:2274 | *OXTR* |  | oxytocin receptor |  |
| Xenbase | Xenopus | Xenbase:XB-GENE-484840 | *oxtr* |  | oxytocin receptor |  |
| ZNC | zebrafish | ZFIN:ZDB-GENE-110805-2 | ***oxtra*** | *oxtr* | **oxytocin receptor a** | oxytocin receptor |
|  |  | ZFIN:ZDB-GENE-110805-1 | ***oxtrb*** | *oxtrl* | **oxytocin receptor b** | oxytocin receptor like |

**Table S9:** Approved nomenclature for the arginine vasopressin receptor 1A genes in vertebrates. Newly approved nomenclature is highlighted in bold.

| **Gene Nomenclature Committee** | **Species** | **Database unique ID** | **Current Approved Symbol** | **Current Approved Name** |
| --- | --- | --- | --- | --- |
| HGNC | human | HGNC:895 | *AVPR1A* | arginine vasopressin receptor 1A |
| MGNC | mouse | MGI:1859216 | *Avpr1a* | arginine vasopressin receptor 1A |
| RGD | rat | RGD:2185 | *Avpr1a* | arginine vasopressin receptor 1A |
| VGNC | chimpanzee, macaque, cat, dog, horse, cow, pig | VGNC:4943, VGNC:70197, VGNC:68838, VGNC:38318, VGNC:15710, VGNC:26358, VGNC:85703 | *AVPR1A* | arginine vasopressin receptor 1A  (**vasopressin receptor 1A** in pig only) |
| CGNC | chicken | CGNC:7451 | *AVPR1A* | arginine vasopressin receptor 1A |
| Xenbase | Xenopus | Xenbase:XB-GENE-482551 | *avpr1a* | arginine vasopressin receptor 1A |
| ZNC | zebrafish | ZFIN:ZDB-GENE-101028-2 | *avpr1aa* | arginine vasopressin receptor 1Aa |
|  |  | ZFIN:ZDB-GENE-041210-105 | *avpr1ab* | arginine vasopressin receptor 1Ab |

**Table S10:** Approved nomenclature for the arginine vasopressin receptor 1B genes in vertebrates. Newly approved nomenclature is highlighted in bold.

| **Gene Nomenclature Committee** | **Species** | **Database unique ID** | **Current Approved Symbol** | **Current Approved Name** | **Previous Approved Name** |
| --- | --- | --- | --- | --- | --- |
| HGNC | human | HGNC:896 | *AVPR1B* | arginine vasopressin receptor 1B |  |
| MGNC | mouse | MGI:1347010 | *Avpr1b* | arginine vasopressin receptor 1B |  |
| RGD | rat | RGD:6502812 | *Avpr1b* | **arginine vasopressin receptor 1B** | vasopressin V1b receptor-like |
| VGNC | chimpanzee, macaque, cat, dog, horse, cow, pig | VGNC:452, VGNC:70198, VGNC:68844, VGNC:38319, VGNC:15711, VGNC:26359, VGNC:85704 | *AVPR1B* | arginine vasopressin receptor 1B  (**vasopressin receptor 1B** in pig only) |  |
| CGNC | chicken | CGNC:505 | *AVPR1B* | arginine vasopressin receptor 1B |  |
| Xenbase | Xenopus | Xenbase:XB-GENE-481094 | *avpr1b* | arginine vasopressin receptor 1B |  |
| ZNC | zebrafish |  | not present |  |  |

**Table S11:** Approved nomenclature for the arginine vasopressin receptor 2 genes in vertebrates. Newly approved nomenclature is highlighted in bold.

| **Gene Nomenclature Committee** | **Species** | **Database unique ID** | **Current Approved Symbol** | **Current Approved Name** | **Previous Approved Name** |
| --- | --- | --- | --- | --- | --- |
| HGNC | human | HGNC:897 | *AVPR2* | arginine vasopressin receptor 2 |  |
| MGNC | mouse | MGI:88123 | *Avpr2* | arginine vasopressin receptor 2 |  |
| RGD | rat | RGD:2186 | *Avpr2* | arginine vasopressin receptor 2 |  |
| VGNC | chimpanzee, macaque, cat, dog, horse, cow, pig | VGNC:1407, VGNC:108053, VGNC:68849, VGNC:38320, VGNC:15712, VGNC:26360, VGNC:97898 | *AVPR2* | arginine vasopressin receptor 2  (**vasopressin receptor 2** in pig only) |  |
| CGNC | chicken |  | not currently annotated in birds |  |  |
| Xenbase | Xenopus | Xenbase:XB-GENE-482287 | *avpr2* | **arginine vasopressin receptor 2** | arginine vasopressin receptor 2 (nephrogenic diabetes insipidus) |
| ZNC | zebrafish | ZFIN:ZDB-GENE-090313-344 | *avpr2aa* | arginine vasopressin receptor 2a, duplicate a |  |
|  |  | ZFIN:ZDB-GENE-110411-48 | *avpr2ab* | arginine vasopressin receptor 2a, duplicate b |  |

**Table S12:** Approved nomenclature for the arginine vasopressin receptor 2B genes in vertebrates. Newly approved nomenclature is highlighted in bold.

| **Gene Nomenclature Committee** | **Species** | **Database unique ID** | **Current Approved Symbol** | **Previous Approved Symbol** | **Current Approved Name** | **Previous Approved Name** |
| --- | --- | --- | --- | --- | --- | --- |
| HGNC | human |  | not present |  |  |  |
| MGNC | mouse |  | not present |  |  |  |
| RGD | rat |  | not present |  |  |  |
| VGNC | chimpanzee, macaque, cat, dog, horse, cow, pig |  | not present |  |  |  |
| CGNC | chicken |  | not present |  |  |  |
| Xenbase | Xenopus |  | not present |  |  |  |
| ZNC | zebrafish | ZFIN:ZDB-GENE-121023-1 | ***avpr2b.1*** | si:dkey-178o16.4 | **arginine vasopressin receptor 2b, tandem duplicate, 1** | si:dkey-178o16.4 |

**Table S13:** Approved nomenclature for the arginine vasopressin receptor 2C and 2l genes in vertebrates. Newly approved nomenclature is highlighted in bold.

| **Gene Nomenclature Committee** | **Species** | **Database unique ID** | **Current Approved Symbol** | **Previous Approved Symbol** | **Current Approved Name** | **Previous Approved Name** |
| --- | --- | --- | --- | --- | --- | --- |
| HGNC | human |  | not present |  |  |  |
| MGNC | mouse |  | not present |  |  |  |
| RGD | rat |  | not present |  |  |  |
| VGNC | chimpanzee, macaque, cat, dog, horse, cow, pig |  | not present |  |  |  |
| CGNC | chicken | CGNC:7225 | ***AVPR2C*** | *AVPR2* | **arginine vasopressin receptor 2C** | arginine vasopressin receptor 2 |
| Xenbase | Xenopus | Xenbase:XB-GENE-1219042 | ***avpr2c*** | *avpr2.2* | **arginine vasopressin receptor 2C** | arginine vasopressin receptor (nephrogenic diabetes insipidus), gene 2 |
| ZNC | zebrafish | ZFIN:ZDB-GENE-070705-429 | *avpr2l* |  | arginine vasopressin receptor 2, like |  |


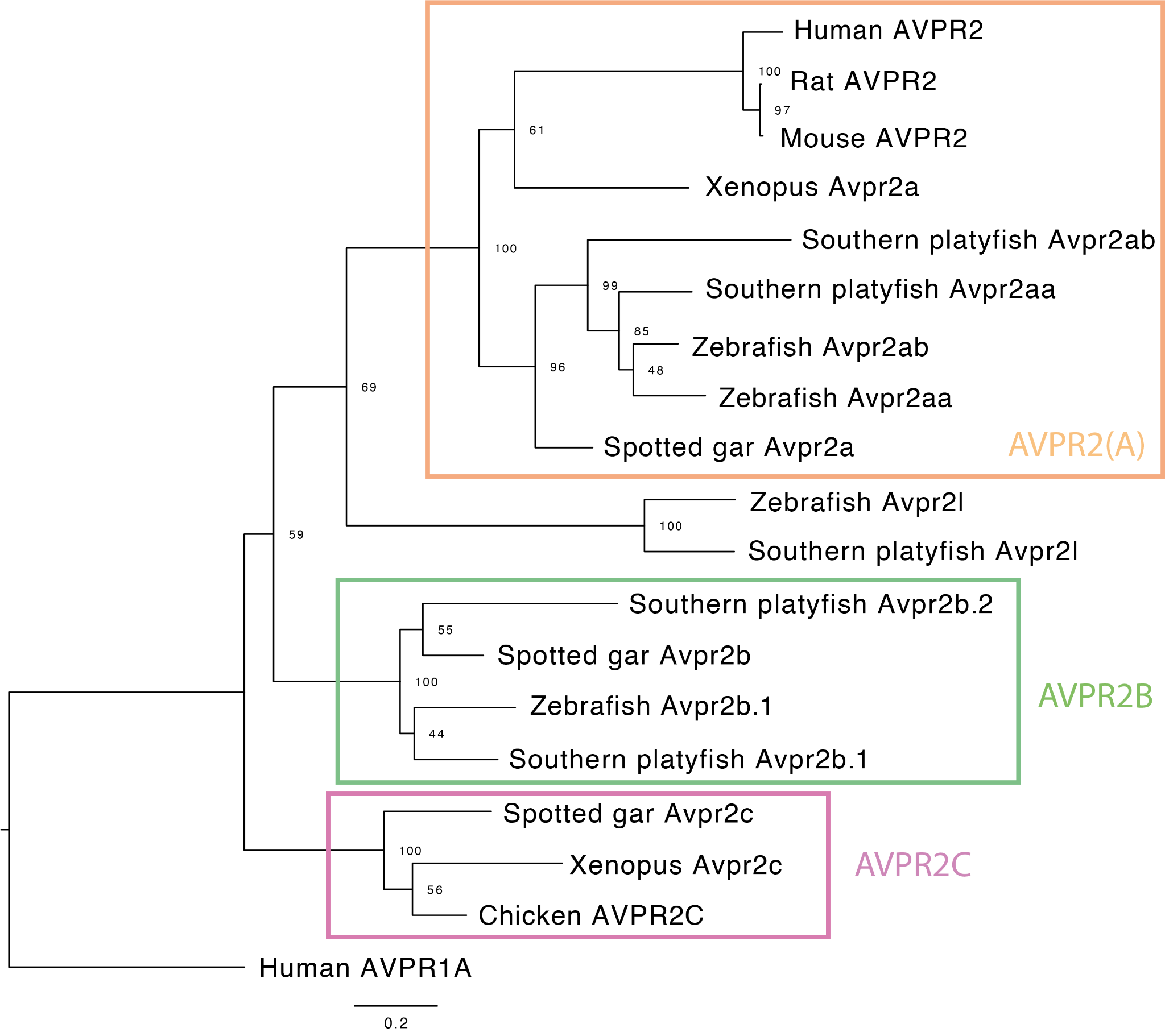


**Figure S1. Maximum likelihood phylogeny of vertebrate AVPR2* amino acid sequences.** The teleost Avpr2l clade does not group with the AVPR2C clade, but also doesn’t group with either the AVPR2(A) or AVPR2B clades with high confidence. Node labels indicate UltraFast Bootstrap [(Minh, Nguyen, and von Haeseler 2013)](https://paperpile.com/c/qR92DB/AtAf) with 1000 replicates, values <95 are not well supported. Human AVPR1A was used as the outgroup. Please note that we do not attempt to resolve the order of divergence of the AVPR2(A), AVPR2B or AVPR2C clades with this analysis.

Methods: Amino acid sequences for AVPR2* were aligned using MUSCLE [(Madeira et al. 2019)](https://paperpile.com/c/qR92DB/hS0U) and trimmed to remove columns with more than 20% gaps using trimAl [(Capella-Gutiérrez, Silla-Martínez, and Gabaldón 2009)](https://paperpile.com/c/qR92DB/rQKi). Maximum likelihood phylogenetic analysis was performed using the IQTree WebServer [(Trifinopoulos et al. 2016)](https://paperpile.com/c/qR92DB/Vg3D) using default parameters. Sequence accession numbers: Human AVPR1A NP_000697.1, Human AVPR2 NP_000045.1, Rat Avpr2 NP_062009.1, Mouse Avpr2 O88721, Chicken AVPR2C NP_001026650.1, Xenopus Avpr2a XP_004916778.1, Xenopus Avpr2c XP_002932869.2, Zebrafish Avpr2aa A0A2R8QNI4, Zebrafish Avpr2ab XP_001922042.4, Zebrafish Avpr2l A5WWC0, Zebrafish Avpr2b.1 E7F1C0, Southern platyfish Avpr2aa XP_023191692.1, Southern platyfish Avpr2ab XP_014325300.2, Southern platyfish Avpr2b.1 XP_005808542.2, Southern platyfish Avpr2b.2 XP_005799961.3, Southern platyfish Avpr2l XP_005799151.1. Spotted gar exonic nucleotide sequences were obtained from [(Theofanopoulou et al. 2021)](https://paperpile.com/c/qR92DB/30ir) and translated using ExPASy translate.

**Supplementary References**

[Bradford, Yvonne M., Ceri E. Van Slyke, Leyla Ruzicka, Amy Singer, Anne Eagle, David Fashena, Douglas G. Howe, et al. 2022. “Zebrafish Information Network, the Knowledgebase for Danio Rerio Research.” *Genetics* 220 (4). https://doi.org/](http://paperpile.com/b/qR92DB/OtYA)[10.1093/genetics/iyac016](http://dx.doi.org/10.1093/genetics/iyac016)[.](http://paperpile.com/b/qR92DB/OtYA)

[Brownstein, M. J., J. T. Russell, and H. Gainer. 1980. “Synthesis, Transport, and Release of Posterior Pituitary Hormones.” *Science* 207 (4429): 373–78.](http://paperpile.com/b/qR92DB/Jxn1)

[Capella-Gutiérrez, Salvador, José M. Silla-Martínez, and Toni Gabaldón. 2009. “trimAl: A Tool for Automated Alignment Trimming in Large-Scale Phylogenetic Analyses.” *Bioinformatics*  25 (15): 1972–73.](http://paperpile.com/b/qR92DB/rQKi)

[Kusumi, Kenro, Rob J. Kulathinal, Arhat Abzhanov, Stephane Boissinot, Nicholas G. Crawford, Brant C. Faircloth, Travis C. Glenn, et al. 2011. “Developing a Community-Based Genetic Nomenclature for Anole Lizards.” *BMC Genomics* 12 (November): 554.](http://paperpile.com/b/qR92DB/g9VG)

[Land, H., G. Schütz, H. Schmale, and D. Richter. 1982. “Nucleotide Sequence of Cloned cDNA Encoding Bovine Arginine Vasopressin-Neurophysin II Precursor.” *Nature* 295 (5847): 299–303.](http://paperpile.com/b/qR92DB/7jb96)

[Madeira, F., Y. M. Park, J. Lee, N. Buso, T. Gur, N. Madhusoodanan, P. Basutkar, et al. 2019. “The EMBL-EBI Search and Sequence Analysis Tools APIs in 2019.” *Nucleic Acids Research* 47 (W1): W636–41.](http://paperpile.com/b/qR92DB/hS0U)

[Malatesta, Marco, Giulia Mori, Domenico Acquotti, Barbara Campanini, Alessio Peracchi, Parker B. Antin, and Riccardo Percudani. 2020. “Birth of a Pathway for Sulfur Metabolism in Early Amniote Evolution.” *Nature Ecology & Evolution* 4 (9): 1239–46.](http://paperpile.com/b/qR92DB/BJvw)

[Minh, Bui Quang, Minh Anh Thi Nguyen, and Arndt von Haeseler. 2013. “Ultrafast Approximation for Phylogenetic Bootstrap.” *Molecular Biology and Evolution* 30 (5): 1188–95.](http://paperpile.com/b/qR92DB/AtAf)

[Ocampo Daza, Daniel, Christina A. Bergqvist, and Dan Larhammar. 2021. “The Evolution of Oxytocin and Vasotocin Receptor Genes in Jawed Vertebrates: A Clear Case for Gene Duplications Through Ancestral Whole-Genome Duplications.” *Frontiers in Endocrinology* 12: 792644.](http://paperpile.com/b/qR92DB/KjhK)

[Ocampo Daza, Daniel, Michalina Lewicka, and Dan Larhammar. 2012. “The Oxytocin/vasopressin Receptor Family Has at Least Five Members in the Gnathostome Lineage, Inclucing Two Distinct V2 Subtypes.” *General and Comparative Endocrinology* 175 (1): 135–43.](http://paperpile.com/b/qR92DB/5caD)

[Theofanopoulou, Constantina, Gregory Gedman, James A. Cahill, Cedric Boeckx, and Erich D. Jarvis. 2021. “Universal Nomenclature for Oxytocin-Vasotocin Ligand and Receptor Families.” *Nature* 592 (7856): 747–55.](http://paperpile.com/b/qR92DB/30ir)

[Trifinopoulos, Jana, Lam-Tung Nguyen, Arndt von Haeseler, and Bui Quang Minh. 2016. “W-IQ-TREE: A Fast Online Phylogenetic Tool for Maximum Likelihood Analysis.” *Nucleic Acids Research* 44 (W1): W232–35.](http://paperpile.com/b/qR92DB/Vg3D)

[Tweedie, Susan, Bryony Braschi, Kristian Gray, Tamsin E. M. Jones, Ruth L. Seal, Bethan Yates, and Elspeth A. Bruford. 2021. “Genenames.org: The HGNC and VGNC Resources in 2021.” *Nucleic Acids Research* 49 (D1): D939–46.](http://paperpile.com/b/qR92DB/jo19)

[Yamaguchi, Yoko, Hiroyuki Kaiya, Norifumi Konno, Eri Iwata, Mikiya Miyazato, Minoru Uchiyama, Justin D. Bell, et al. 2012. “The Fifth Neurohypophysial Hormone Receptor Is Structurally Related to the V2-Type Receptor but Functionally Similar to V1-Type Receptors.” *General and Comparative Endocrinology* 178 (3): 519–28.](http://paperpile.com/b/qR92DB/6ER3)

[Yates, Bethan, Bryony Braschi, Kristian A. Gray, Ruth L. Seal, Susan Tweedie, and Elspeth A. Bruford. 2017. “Genenames.org: The HGNC and VGNC Resources in 2017.” *Nucleic Acids Research* 45 (D1): D619–25.](http://paperpile.com/b/qR92DB/iyVj)
